# Community sensitization and decision‐making for trial participation: A mixed‐methods study from The Gambia

**DOI:** 10.1111/dewb.12160

**Published:** 2017-08-16

**Authors:** Susan Dierickx, Sarah O'Neill, Charlotte Gryseels, Edna Immaculate Anyango, Melanie Bannister‐Tyrrell, Joseph Okebe, Julia Mwesigwa, Fatou Jaiteh, René Gerrets, Raffaella Ravinetto, Umberto D'Alessandro, Koen Peeters Grietens

**Keywords:** community sensitization, informed consent, sub‐Saharan Africa, The Gambia, trial participation, medical research

## Abstract

**Background:**

Ensuring individual free and informed decision‐making for research participation is challenging. It is thought that preliminarily informing communities through ‘community sensitization’ procedures may improve individual decision‐making. This study set out to assess the relevance of community sensitization for individual decision‐making in research participation in rural Gambia.

**Methods:**

This anthropological mixed‐methods study triangulated qualitative methods and quantitative survey methods in the context of an observational study and a clinical trial on malaria carried out by the Medical Research Council Unit Gambia.

**Results/discussion:**

Although 38.7% of the respondents were present during sensitization sessions, 91.1% of the respondents were inclined to participate in the trial when surveyed after the sensitization and prior to the informed consent process. This difference can be explained by the informal transmission of information within the community after the community sensitization, expectations such as the benefits of participation based on previous research experiences, and the positive reputation of the research institute. Commonly mentioned barriers to participation were blood sampling and the potential disapproval of the household head.

**Conclusion:**

Community sensitization is effective in providing first‐hand, reliable information to communities as the information is cascaded to those who could not attend the sessions. However, further research is needed to assess how the informal spread of information further shapes people's expectations, how the process engages with existing social relations and hierarchies (e.g. local political power structures; permissions of heads of households) and how this influences or changes individual consent.

## 1 BACKGROUND

Individual informed consent is an essential ethical requirement and standard procedure to all research involving human participants. ‘Respect for persons’, one of the internationally agreed ethical principles, stipulates that potential participants should have (i) access to sufficient information; (ii) competence, i.e. having the capacity to understand that information; (iii) free choice, decision making in the absence of coercion or deception; and, (iv) comprehension of the subject.^1^ Thus, participation in research requires individuals to be in a position to freely give informed consent after the individual consent interview, in which all relevant information is provided, discussed and understood by the potential participant.

However, research shows that translating the principle of ‘respect for persons’ into practice is often challenging.^2^ Though some challenges are ubiquitous, the ethical pitfalls are more salient in resource‐limited settings.^3^ In some of these settings, participation in health research may be the only way to obtain (free or better quality) health services; furthermore, high levels of illiteracy may hamper the comprehension of informed consent.^4^

In order to improve potential study participants’ comprehension of research, community level meetings prior to the start of the research have been recommended.^5^ Community level consultations have been implemented by the Medical Research Council Unit Gambia (MRCG) in the form of community sensitizations, which is a process whereby research staff arrange meetings to make information on the research available in the villages from which potential research participants may be recruited. All villagers are invited to a central meeting space where the study is explained and questions can be addressed. At the end of this process, permission to carry out the research in the village may be given by the *Alkalo* (village leader) or a representative in case of his absence. Although such permission cannot, in any way, replace individuals’ consent, it represents the preliminary agreement of the community.

This study aimed to assess the relevance of community sensitization for individual decision‐making and to identify additional factors influencing people's participation in research in rural Gambia.

## 2 METHODS

### 2.1 Study site and population

This mixed‐methods study was part of the anthropological research conducted within the framework of two different malaria studies carried out by the MRCG in rural Gambia (Table 1). The mixed‐methods study was carried out between May 2013 and November 2014 in 21 rural communities in all five regions of The Gambia (i.e. West Coast River, North Bank, Lower River, Central River and Upper River Regions). Study participants were from different ethnic groups, mainly *Mandinka, Jahanka, Serahule, Wolof, Jola* and *Fula*. The large majority are Muslim.

**Table 1:** Study site, population and design of Malaria Transmission Dynamics and Prinogam

	Malaria Transmission Dynamics	Prinogam
Participants screened	4500	7319
Number of fieldworkers	7 (including a supervisor)	8 (including a supervisor)
Number of field nurses	7 (including a supervisor)	6 (including a supervisor)
Movement of field staff	Resided in the study villages during the rainy season	Field staff moved from village to village during peak of malaria season
Objective of the study	Understanding the dynamics of malaria transmission and at factors determining its heterogeneity at village level	Evaluation of the effect of different primaquine regimens to clear gametocyte carriage
Summary of procedure	Community sensitization meeting in each village.The field staff made individual visits to compounds directly after the community meetings to discuss the study in more detail and to carry out the individual informed consent. Children between 10‐18 years were asked to provide an assent, in addition to the parents’ consent; for children <10 years old, there was only the parents, informed consent.The study participants were actively followed up during the rainy season by monthly bleedings carried out by field nurses and passive case detection at local health facilities. The field nurses collected a blood sample for the detection of malaria infections by PCR (polymerase chain reaction), and in case of symptomatic individuals an RDT for the immediate detection of a malaria infection was performed. In addition, a monthly survey was carried out by the fieldworkers.Each year, the community sensitizations were repeated in order to discuss the study with villagers and inform about new procedures.In 2014‐2015, a mass drug administration was carried out in the beginning of the rainy season.	Community sensitization meeting in each village.The field staff moved from compound to compound and verbal consent was taken for a rapid clinical assessment and an RDT.Blood from a single finger prick from all non‐febrile people was used to prepare a blood slide and perform an RDT. If the RDT was positive, the blood slide was taken to the laboratory to determine the parasite density. Participants with a *Plasmodium falciparum* mono‐infection with a density of at least 20 parasites/μL were informed of the result and invited to the clinic the following day. After obtaining the written informed consent, a finger prick blood sample was collected for haemoglobin (Hb) measurement and G6PD (Glucose‐6‐phosphatase dehydrogenase) screening. If eligible, participants were referred to the trial clinician for randomisation and enrolment. Each participant was followed up on days 1, 2, 3, 7, 10, 14, 21, 28, 35, and 42 or on any other day outside scheduled visits if they felt sick. At each visit, a blood sample (about 0.5ml by fingerprick) was collected for the determination of gametocytaemia, parasite clearance and Hb.In addition, a venous blood sample (3ml) was collected from a subset of 100 participants that consented for a direct membrane feeding assay to determine infectiousness to mosquitoes on day 7.

The Gambia is one of the smallest and poorest African countries.^6^ In this resource‐poor context, most families’ livelihood is based on the farming of groundnuts and maize, small‐scale informal trade and remittances from relatives that have migrated to urban areas of the country and abroad.^7^ Treatment seeking itineraries are characterized by medical pluralism. Access to health care varies greatly between study villages, but is overall more challenging on the North Bank due to structural factors such as the distance to the health center or hospital, transport options and road infrastructure. Despite the common perception that the health center is the best place to receive treatment, adults and elderly people often delay visits or rely on home treatment.

### 2.2 The Malaria Transmission Dynamics study and Prinogam malaria treatment trial

This mixed‐method study was ancillary to two malaria studies carried out by the MRCG in rural Gambia (Table 1). These two studies were selected because they (i) involved community sensitization meetings preceding the start of recruitment; (ii) were similar in terms of organization, profile of fieldworkers and target population; and, (iii) they contained anthropological work packages that included long‐term ethnographic research carried out by the same researchers.^8^

#### 2.2.1 Malaria Transmission Dynamics study

The first study was an observational study to determine the dynamics of malaria transmission in different sites across the country (*Malaria transmission dynamics in The Gambia: Defining the spatial and temporal spread of malaria at micro‐level (village);* referred to hereafter as the “Malaria Transmission Dynamics” study) (Table 1).^9^ Community sensitization meetings were organized in all 12 study villages. The study targeted all community members older than 6 months residing in the study villages. Minors between 10‐18 years old were asked to provide assent in addition to the parents’ consent; both agreements were necessary for participation. For children <10 years of age, only the parents’ informed consent was needed. The informed consent interview was conducted in the local language, based on an English written consent form and information sheet. The study participants were actively followed up during the rainy season by monthly blood sampling carried out by field nurses and passive case detection at local health facilities. In addition, a monthly survey was carried out by the fieldworkers. As part of this study, a mass drug administration campaign was carried out in a later phase.

#### 2.2.2 Prinogam

The second study was a randomized controlled trial carried out in the Central and Upper River Regions of The Gambia on the efficacy of different doses of primaquine in clearing gametocytes in asymptomatic malaria infected individuals (*Primaquine's gametocytocidal efficacy in malaria asymptomatic carriers treated with dihydroartemisinin‐piperaquine*; ClinicalTrials.gov: NCT01838902; referred to hereafter as “Prinogam”) (Table 1).^10^ As part of *Prinogam*, community sensitization meetings were held in each village. A couple of days after meeting the community, the field staff moved from compound to compound and verbal consent was requested for a rapid clinical assessment and a Rapid Diagnostic Test (RDT). At this stage all people above the age of one, and weighing more then 10 kg could be included. Blood from a single finger prick from all non‐febrile people was used to prepare a blood slide and perform an RDT. If positive, the blood slide was taken to the laboratory to confirm the infection and estimate the parasite density. Patients were further recruited in the study if they consented to participation after undergoing the full informed consent interview. The interview was conducted in the local languages, based on the English written consent form and information sheet of the study, or with the support of an ad hoc multimedia tool in the local languages as part of a nested trial.^11^

### 2.3 Study design

The anthropological study used a sequential mixed‐methods design comprising qualitative and quantitative methods.^12^ Qualitative research was carried out in both studies in order to get an in‐depth understanding of the research context. Qualitative research was carried out before, during and after the community sensitization in the context of the *Malaria Transmission dynamics* and *Prinogam* studies. Explorative research was carried out before the community sensitization in order to (i) develop and pre‐test the questionnaire for the second strand and (ii) to understand the research context. Qualitative research continued during and after the community sensitization. The quantitative study was carried out in the context of the *Prinogam* study. Quantitative data on study participation was not collected within the *Malaria Transmission Dynamic* study as this study requested monthly testing and treatment and villagers had multiple opportunities to enroll in, or step out of, the study.

### 2.4 Qualitative research

#### 2.4.1 Data collection

Data were collected using individual interviews, group discussions and participant observation.

*In‐depth interviews* were based on question guides focusing on a broad range of possible factors potentially influencing people's decision‐making regarding trial participation such as the informed consent procedures, social relationships, the meaning of blood and perceptions regarding the MRCG. Interviews were carried out with community members to assess their attendance during the community sensitization, their intention to participate and their perceptions regarding the MRCG. Interviews were recorded and fully transcribed. When it was not possible and/or inappropriate to record the interviews, conversations with informants were written down during or immediately after the interview. Interviews were mostly carried out by the researchers and their research assistants in English or in one of the local languages.

*Group discussion* were carried out when informants agreed to be interviewed together, for example, women who were looking after children in the same compound, men chatting in the village *bantaba* (central meeting place).

*Participant observation*. The researchers and fieldworkers repeatedly visited and stayed in the study villages for a few days at a time. During these stays, people's everyday routines were observed and informal conversations about relevant themes were held with adult community members, which were written down after the interview as soon as appropriate. The long‐term engagement with the study villages facilitated trust between the social science research team and the study participants enabling to discuss more sensitive topics such as, for example, fears of trial participation and other barriers such as conceptions of blood relevant to blood sampling.

#### 2.4.2 Sampling

Sampling was theoretical (i.e. in accordance with emerging results from the data collection in order to contribute to theory building).^13^ Informants were selected from both study sites. People were interviewed regardless of their attendance to the community meeting and their individual decision to participate in the malaria studies. Informants were categorized according to relevant criteria (e.g. gender, profession, village) in order to obtain maximum variation. In addition, snowball sampling techniques (i.e. where participants identify additional respondents) were used.^14^ This technique was particularly useful when it concerned (i) more sensitive topics such as perceptions regarding blood whereby the referral by somebody within their social network increased the new respondent's trust in the research team, and (ii) made it possible to contact people who were hard to reach, such as *marabouts* (traditional healers). Infants or children below the age of 14 were not included in our sample since different informed consent procedures were used (assent from parents or guardian in addition to informed consent).

#### 2.4.3 Analysis

Data analysis was concurrent to data collection. Preliminary data from the interviews, group discussions and observations were analyzed to inform the interview and observation guide; additional data was consequently collected to confirm or refute temporary results until saturation was reached. Interviews were systemized and analyzed with NVivo Qualitative Analysis software (QSR International Pty Ltd. Cardigan UK). Quotes were selected for this manuscript to illustrate main themes.

### 2.5 Quantitative Research

#### 2.5.1 Data collection

A cross‐sectional survey with open and closed questions was carried out in the context of the *Prinogam* study. The questionnaire was administered *after* the *Prinogam* community sensitizations but *before* the field staff moved from compound to compound to obtain verbal consent for the Prinogam study's rapid clinical assessment to detect malaria infection. The questionnaire was administered by trained interviewers in the privacy of the compound. The questionnaire assessed participants’ (i) presence during community sensitization meetings; (ii) reasons for their absence/presence; (iii) inclination to participate *before* the individual informed consent; (iv) previous research experiences with the MRCG; (v) views of the MRCG; and, (vi) socio‐demographic characteristics.

#### 2.5.2 Sampling

Villages are composed of compounds consisting of households that are arranged according to patrilineal lineages. The sampling frame comprised all 124 compounds belonging to the 9 study villages that were purposefully selected to participate in the mixed‐methods study during the *Prinogam* screening period (August – December 2013) (total inhabitants: 4456 people). In each compound, the compound head (the oldest married man) and often several household heads, i.e. some of the married brothers, nephews and sons of the compound head reside together with their wives and children. Authority figures (compound heads) within each compound were then selected to respond to the questionnaire as the exploratory qualitative research had indicated their principal role in decision‐making processes affecting the compound.

When compound heads were not available during the visit of the interviewer, the next person in the internal hierarchy was selected, which in most cases was a household head or spouse.

#### 2.5.3 Analysis

Quantitative data was double entered in EpiData 3.1. (CDC, Atlanta; WHO Geneva, 1996) and cleaned and analyzed in SPSS (IBM SPSS Statistics 22). Frequency tables and cross‐tabulations for the main outcome variables were produced.

### 2.6 Ethical clearance

This social science study was approved by the MRC (Medical Research Council) Scientific Coordinating Committee and the Gambia Government/MRC joint Ethics Committee (SCC number 1319; 1351); and by the Institutional Review Board of the Institute of Tropical Medicine, Antwerp, Belgium. The interviewers followed the Code of Ethics of the American Anthropological Association (AAA). All interviewees were informed before the start of the interview about project goals, the topic and type of questions as well as their right to decline participation or to interrupt the conversation at any time. Verbal instead of written consent was preferred as requesting the subject's signature could have been a potential reason for mistrust. All original recordings, transcriptions and notes were anonymized and access to the social science database was restricted to members of the research team.

## 3 RESULTS

### 3.1 Study participants

#### 3.1.1 Qualitative strand

In‐depth interviews (n=238) and informal conversations (n=17) were carried out with community members and field staff (Table 2, 3). Group discussions (n=9) were conducted when informants agreed to be interviewed together, for example, women who were looking after children in the same compound, men chatting in the village *bantaba* (central meeting place) (Table 4). In addition, on several occasions observations were done by the research team and recorded in field notes. The research team observed (i) community sensitization meetings in eight different villages; (ii) reactions to the *Prinogam* trial at community level and at the health center; and, (iii) daily events in the study villages.

**Table 2:** Socio‐demographic characteristics respondents in‐depth interviews

	MTD (n)	Prinogam (n)	Total
**Profession**			
Policeman	0	1	1
Midwife	2	0	2
Blacksmith	0	2	2
Weaver	0	2	2
Imam	1	2	3
Griot	1	2	3
Retired	0	3	3
Teacher	2	2	4
Trader	0	4	4
Community health worker	3	2	5
Traditional birth attendant	0	9	9
Herder	0	10	10
Traditional healer	1	10	11
Alkalo	6	7	13
Farmer	48	150	198
**Gender**			
Men	35	95	130
Women	29	80	109
**Age category**			
Teenager	0	8	8
Adult	35	126	161
Elder	29	40	69
**Total**	64	174	238

**Table 3:** Socio‐demographic characteristics respondents informal conversations

	MTD (n)	Prinogam (n)	Total
**Profession**			
Alkalo	0	2	2
Businessman	1	0	1
Migrant	0	1	1
Farmer	3	0	3
Housewife	0	1	1
Hunter	1	0	1
Traditional healer	0	1	1
MRCG driver	0	1	1
MRCG fieldworker	0	5	5
MRCG reporter	0	1	1
**Gender**			
Men	2	11	13
Women	3	1	4
**Total**	5	12	17

**Table 4:** Overview compositions groups during group discussions

Profession	Gender	Age category	Study
Farmers	Women	Adults (children present)	MTD
Farmers	Women and men	Elders and adults (with children present)	MTD
Farmers	Women and men	Adults	MTD
Civil servant and taxi driver	Men	Adult	MTD
MRC Fieldworkers	Men	Adult	Prinogam
Farmers	women	Adults	Prinogam
Farmers	Women and men	Adults	Prinogam
Herders	Men	Adults	Prinogam
Farmers	Women and men	Adults	Prinogam

#### 3.1.2 Quantitative strand

In total 124 questionnaires were completed; there were no refusals. There were slightly more men (52.4%) than women (47.6%) (Table 5). Most respondents were not able to read or write (66.9%). The main ethnic groups included were *Fula* (44.4%), *Jahanka* (39.5%), *Madinka* (14.5%) and *Serahule* (1.6%). The median age of respondents was 48.5 years old (q1: 39 years; q3: 65 years). 37,1% had previously participated in MRCG research.

**Table 5:** Socio‐demographic characteristics respondents quantitative study (Prinogam) (N=124)

Gender	n	%
Male	65	52.4
Female	59	47.6
**Position within household**		
Compound head/household head	58	46.8
Mother/wife	50	40.3
Son	9	7.3
Daughter	3	2.4
Other	4	3.2
**Ethnicity**		
Fula	55	44.4
Jahanka	49	39.5
Madinka	18	14.5
Serahule	2	1.6
**Age categories**		
19 – 30	19	15.3
31 – 40	22	17.8
41 – 50	29	23.4
51 – 60	18	14.5
60 +	36	29
Median age	48.5	/
**Knows how to read and write**		
No	83	66.9
Yes	41	33.1
**Previously participated in MRCG study**		
Did not participate in MRCG study	78	62.9
Did participate in MRCG study	46	37.1

### 3.2 Community sensitization procedures

#### 3.2.1 The role of fieldworkers and the structure of community sensitization meetings

In both studies, the MRCG field team consisted of fieldworkers and nurses. Most fieldworkers and nurses assisting in the community sensitizations had worked for previous MRCG research projects and all had followed training in research ethics and Good Clinical Practices in addition to receiving training on consent procedures within the specific studies. In both studies, fieldworkers and nurses were at the interface between the MRCG and community members, both during the community sensitization and individual informed consent process. The community sensitization meetings were held in the main *bantaba* (traditional meeting place) of all villages included in both studies. The field team often decided to work together with key community members, pointed out by the village leader (*Alkalo)* or a representative, such as the community health worker or praise‐singers *(griots)*, to invite and assemble the inhabitants of the village. Towards the end of the meeting, up to three kilograms of kola nuts were given to the *Alkalos* of the communities to share with the villagers. Kola nuts are customary items for meetings in villages and signify a traditional token of respect. Over the course of these community sensitization meetings, which lasted 1h‐1h30, there were several people who left before the meeting finished.

#### 3.2.2 Communication during sensitization meetings

The qualitative research demonstrated that both research staff and community members considered community sensitization procedures important to the success of the study (Table 6). While the information given during the community sensitization meetings was consistent with the information written down in the English written consent form and information sheet, the MRCG team explained the study by giving concrete examples, making drawings in the sand and by relating to people through jokes. In both studies, they stressed the utility of research in general and the need to test whether medical products are effective by providing examples from previous research carried out by the MRCG. The value of research for the whole country was also frequently stressed (Table 6). Personal as well as community benefits were mentioned – such as the presence of a doctor and the availability of free malaria medication (*Malaria Transmission Dynamic* study). Possible risks of the research and frequent concerns about blood sampling by the local population were also addressed (*Malaria Transmission Dynamic* study and *Prinogam)* (Table 6). They consistently explained that respondents should take their decision voluntarily and that all personal information would be kept confidential (Table 6). In both studies, the villagers were encouraged to ask questions and share ideas about the research project. Observational data during the community sensitization meetings of the *Malaria Transmission Dynamics* study indicated that, in line with cultural norms on authority, mainly male and female elders asked questions, after which the *Alkalo* gave his approval for the study in the village.

**Table 6:** Illustrative quotes on communication during community sensitization meeting

Quote regarding the importance of community sensitization	Data source	Informant	Study
According to the people of village X, the *Alkalo* of village Y is very harsh to his own people. He is a divide and rule form of ruler. He is always in trouble with his own people in the village. Even the program that MRC brought to the village, the *Alkalo* wants to create a problem, to boycott the program due to his personal feelings without considering the interest of the village as a responsible leader. He even convinced his own relatives not to join the MRC program because he told them the MRC is always involved in blood taking which has a big impact on our health by weakening our body. But thanks to the good people of the village, most especially the youths and some village elders, who have played a vital role in convincing the people to participate in the MRC program with or without the *Alkalo*. This was based on the sensitization made by the MRC people, which has been fully appreciated by the community.	Informal conversation	Farmer	Malaria Transmission Dynamics
**Quotes regarding benefits of research**			
There is a high degree of hope that when you take that medicine, from the time that you have taken it to the end of the rainy season, you won't encounter the problem of malaria, but this cannot be proven now. How can we put this to prove? It's only through testing that we can prove this. That is if we test it over and over.	Community sensitization	Fieldworker	Malaria Transmission Dynamics
You see this Coartem, the new medicine that you are seeing here, we were the first people who made a research on it in the Gambia. Now the whole country is using it. So, when we know the medication that is introduced now, would be beneficial, then we can add it onto our treatment facilities. This is what would be the *toubabs* (white people) pride. If there is any benefit for the *toubabs*, then that is the benefit.	Community sensitization	Fieldworker	Malaria Transmission Dynamics
**Quote regarding side effects**			
The new medicine is just like Paracetamol or chloroquine or any other medicine… In the sense that when you take it, it can cause dizziness or high body temperature because the body is not used to it. It can also give you a little stomach disorder, but apart from that, it brings no other problem.	Community sensitization	Fieldworker	Malaria Transmission Dynamics
**Quotes regarding blood samples**			
It can be that you are not feeling sick during the rainy season but that you are sick. We will do a blood prick on filter paper. The MRC has a machine to test and see if you have malaria or not. We will count the malaria parasites. We want to know why some people are stronger against malaria? Why is malaria aggressive for some and not for others?	Community sensitization	Fieldworker	Malaria Transmission Dynamics
Some will claim that MRC is here to withdraw your blood. The ways in which blood is withdrawn is known to all… they collect your blood and put it in a plastic bag and tell you to give it to the person who needs it. But this one is just blood checking and if anybody asks, tell them that this is blood checking. We are here to check whether you have malaria or not, we are not here to withdraw your blood.	Community sensitization	Fieldworker	Malaria Transmission Dynamics
R: I can now see how some people of MRC are misquoted. Other people like saying that the MRC are taking blood, and they take their blood. But a little amount of blood is not enough for a person to sell. People are misquoted, it is not right.*I: Do people think that the blood sample will be used for witchcraft, the devil or bad eye?*R: I don't see anything true in that. The little blood will not be enough. It is just a sample.*I: it is a common belief of people?*R: No, if people suspect it, the MRC will not even be able to hold a community meeting in this village.	Interview	Farmer	Malaria Transmission Dynamics
**Quotes regarding individual informed consent procedures**			
The most important thing is to say that I will participate willingly because I want to, and not because Mr. X is participating so I will also participate just for the sake of participation. Your participation should be based on something. It has to appeal to your interest and this is why we give you something that the *toubabs* called the consent form.	Community sensitization	Fieldworker	Malaria Transmission Dynamics
It was just about participating in the MRC study. It is not a force. Before you participate in the MRC study they will ask you questions to make things clear to you, they are not there to bother anyone but they are only there to help people. Because even the pricking, they normally only prick the finger and then the drugs will be given to us. The blood that is taken is not much, it is only a little that is collected.	Interview	Housewife/farmer	Prinogam

### 3.3 Attendance during community sensitization

The survey indicated that 38.7% (48/124) of respondents had attended the *Prinogam* meeting (Table 7) and observations and interviews confirm that many community members were not present during the Malaria Transmission Dynamics study meeting (Table 8). Those members of the community who were perceived as in charge of healthcare and research participation (i.e. compound leaders and mothers) tended to go to the community meetings (Table 7). People who were not present at the community sensitization but did participate later on in the *Prinogam* study did not always know or could not recall what the study was about (Table 8). It was unclear what caused this but it might be due to limited comprehension or poor recall.

**Table 7:** Attendance during community sensitization (Prinogam)

	n	%
**Presence during community sensitization (N=124)**		
I was present during community sensitization	48	38.7
I was not present during community sensitization	76	61.3
**Gender of people present during community sensitization (N=48)**		
Male	29	60.4
Female	19	39.6
**Household position of people present during community sensitization (N=48)**		
Compound or household leader	24	50
Mother	18	37.4
Son	3	6.3
Other	3	6.3
**Reason for being present at community sensitization (N=48)**		
The expectation to get medication/health benefits	15	31.3
Perceived duty to be present	12	25
The expectation that MRC will bring me benefits	10	20.8
To understand the project	8	16.7
I am the *Alkalo,* and need to be present	3	6.3
**Reason for not being present at community sensitization (N=76)**		
I was busy working or doing household chores	26	34.2
I travelled	17	22.4
I did not know there was a meeting	15	19.8
I was sick	7	9.2
I send somebody who informed me later	7	9.2
I preferred to have some leisure time	1	1.3
I thought it was only for the elderly	1	1.3
Missing	2	2.6

**Table 8:** Illustrative quotes on attendance during community sensitization

Quotes from trial participants who did not attend the sensitization meeting	Data source	Informant	Study
*I: Were you present during the community sensitization?* R: No. *I: Why were you not present?* R: I was not informed. I was not aware because they did not tell me.	Interview	Compound head/farmer	Malaria Transmission Dynamics
*Interviewer: Did you go to the sensitization meeting that took place in X?* Respondent: No. *I: If you were not there who told you about the study?* R: No one informed me about the meeting. I saw them doing it but I was sick so I could not go there. *I: So you didn't know about the sensitization meeting at all, when you saw the MRC people you just came along?* R: They came and took my blood. After taking my blood they asked me my name and I told them. They gave me a paper, I placed that paper on the table, and they told me to come here tomorrow. *I: Did you know what the study is about?* R: No.	Interview	Housewife/ farmer	Prinogam
*I: Were you at the sensitization meeting that took place in your village?* Respondent: No, I was not present. *I: Did you know who was at the sensitization meeting?* R: I was not in the village, I travelled. *I: Did people talk about it when you came back? Did people talk about the sensitization in the village?* R: Yes. *I: What were they talking about?* R: They did not explain to me. I was just told that the MRC people were here. *I: Did you know what the study is about before coming here today?* R: No, I did not. *I: Did they do the finger prick test this morning or yesterday?* R: They took some yesterday and took some this morning. *I: What did you think then the study was about?* R: No, I don't know. *I: What exactly are you being treated for today?* R: Malaria.	Interview	Farmer	Prinogam
*I: Were you at the sensitization meeting that took place at X?* R: I am not sure that I was present. *I: If you were not at the sensitization meeting then who told you about the study?* R: I just came from the farm and upon my arrival; I was told that a meeting was held here in the village.(…) *I: How did you find out what the study was about?* R: I have no idea.*I: Can you describe to us what exactly you are being treated for?* R: Yes, I can tell you something about that.*I: Something like what?* R: That they are treating us to make us become healthy. *I: And what else?* R: That is all I know.		Farmer	Prinogam

The reasons for attending these meetings were mainly (i) the wish to receive medication or other health benefits (31.3% or 15/48); (ii) the perceived duty to be present (25% or 12/48); (iii) the expectation to receive benefits, without explicit specifications on what this aid entailed (20.8% or 10/48); and, to understand the project (16.7% or 8/48) (Table 7). Common reasons for not attending the meeting were (i) being too busy working on fields or doing household chores (34.2% or 26/76); (ii) travelling (22.4% or 17/76); or (iii) not having been informed about the meeting (19.8% or 15/76) (Table 7).

### 3.4 Awareness about medical studies

Although only 38.7% of all respondents were present at the community sensitization meetings of *Prinogam* (Table 7), 92.7% of all respondents were aware that the MRCG was planning a study in their village when surveyed *after* the community meeting and *before* individual screening for study (Table 9). This to some extent expected discrepancy can be explained by the MRCG fieldworkers encouraging people to spread the news about the study and community members’ active strategy to send representatives of the compound to the community sensitization meeting to inform others afterwards (Table 10). The survey showed that 9.2% of respondents who did not attend the meeting reported that they had sent somebody else to the meeting to obtain information (Table 7). People developed this strategy since (i) they thought it was important that their compound was represented during all types of community meetings, and (ii) because certain people did not have time to attend the community meetings due to livelihood activities but still wanted to be informed (Table 10).

**Table 9:** Awareness about MRCG study and desire to participate before the individual informed consent procedure (Prinogam) (N=124)

	n	%
**Do you know the MRCG wants to do a study here?**		
Yes	115	92.7
No	9	7.3
**Degree of awareness about MRCG study**		
Completely unaware (did not go to sensitization and did not know about study)	9	7.3
Partly aware (did not go to sensitization but knew about study)	67	54
Aware (went to sensitization and knew about study)	48	38.7
**Would you like to participate in this study by MRCG?**		
Yes	113	91.1
No	6	4.8
It depends	5	4.1

**Table 10:** Illustrative quotes on awareness about medical studies

Quotes on trial awareness	Data source	Informant	Study
In our Fula community (the sensitization is organized in a predominantly Fula community), the number of people available at this point is enough for the meeting. Follow the example of the *Alkalo*, he gives information. Let's start the meeting. The information spread to the people available at this meeting, will later be disseminated to the other people absent in the meeting.	Community sensitzation	Fieldworker	Malaria Transmission Dynamics
Almost everybody from the compound was at the community meeting. Six people were absent because they were in the bush. These were his son and his junior brothers (…) They went farming. The reason I was present during the meeting was so that I could explain everything and whatever I accept, they will follow.	Interview	Compound head/farmer	Malaria Transmission Dynamics
People will talk about the MRC study. It is the new agenda. By now it will be on the ‘word radio’.	Informal conversation	Compound head/farmer	Malaria Transmission Dynamics
I do understand the most important of information said during the community sensitization meeting, not everything. (…)*I: What is the general reaction of the village on the community meeting?* R: People in the village talked about it yesterday. The general reaction is positive. The *Alkalo* is very happy though they are not educated so they might not understand everything, but the people who came and explained it to them did a very good job. *I: Would you like to participate in the study the MRC is about to start?* R: Yes, I am the head. *I: What do you need to do as head?* R: I will help people to better understand the village. I will also explain to those who might misunderstand what the meeting was all about.	Interview	Housewife/farmer	Malaria Transmission Dynamics

### 3.5 Intention to participate

Overall, almost all (91.1%) respondents stated they wanted to participate in the *Prinogam* study although recruitment and the individual informed consent procedure had not yet started (Table 9). When differentiating between levels of awareness, both qualitative and quantitative data indicated that respondents who went to the community meetings were more interested in participating (95.8% (46/48)) than those who were completely unaware of the study (55.6% (5/9) (i.e. did not go to community sensitization meeting and did not hear information about the study) (Table 11). Respondents who were completely unaware of the study could not make their decision regarding research participation at that point in time based on the content of the study since they hadn't heard about the study. Respondents’ intentions regarding research participation was, at the time of the survey, largely based on factors that were not linked to the specific malaria studies but on what they knew about the general benefits and disadvantages of participating in research in general. Furthermore, their perception of the research institution was based on previous studies or word of mouth.

**Table 11:** Willingness to participate compared between levels of awareness before individual informed consent procedure (Prinogam)

	n		%
**Group 1: People completely unaware of the MRCG study (N=9)**			
Yes, would like to participate	5	55.6	
No, would not like to participate	3	33.3	
It depends	1	11.1	
**Group 2: People partly aware of the MRCG study (did not go to sensitization but did know about MRCG study) (N=67)**			
Yes, would like to participate	62	92.5	
No, would not like to participate	2	3	
It depends	3	4.5	
**Group 3: People aware of the MRCG study (went to sensitization) (N=48)**			
Yes, would like to participate	46	95.8	
No, would not like to participate	1	2.1	
It depends	1	2.1	

### 3.6 Perceived benefits and barriers to research participation

#### 3.6.1 Reasons to participate

The MRCG was generally described as ‘*good’;* (40.3%) and seen as improving health (21.8%) or giving benefits (21.8%) (Table 12). During the interviews, nobody explicitly described the main mandate, i.e. research. Hence, the main reason why people were interested in participating was the expectation to receive good health care and medication (59.3%) (Table 13). Qualitative research confirmed that joining MRCG studies was associated with several benefits at the individual level, such as free treatment, transport or payment for transportation to health centers or the MRCG field station in case of illness, and getting examined by qualified MRCG field nurses and clinicians (Table 14). These individual benefits were highly valued given the limited access to other health care providers. People were very well aware that these benefits were linked to participation in the studies.

**Table 12:** Description of the MRCG (Prinogam) (N=124)

	n	%
The MRCG is good	50	40.3
The MRCG helps you with health	27	21.8
The MRCG gives you benefits	27	21.8
The MRCG gives you free things	7	5.6
The MRCG takes blood	3	2.4
The MRCG helps you with malaria	2	1.6
I don't know	8	6.5

**Table 13:** Given reasons for willingness to participate in trial before individual informed consent procedure (Prinogam) (N=113)

	n	%
I think they will bring good health care/medication	67	59.3
I think they will bring some benefits (e.g. knowledge, free things)	20	17.7
The MRCG is a good organization	12	10.6
I want to know if I am healthy	3	2.7
They will help us with malaria	3	2.7
Because somebody I know was cured by the MRCG in the past	3	2.7
The perceived social duty to participate	2	1.7
I am sick	2	1.7
In order to support the government^a^	1	0.9

a Misunderstanding by respondent: participation in this project did not imply that the government was supported.

**Table 14:** Illustrative quotes on perceived benefits and barriers to research participation

Quotes on benefits of research participation	Data source	Informant	Study
The work of MRC is very good. They brought me all the way to Fajara to help me (…). They picked me up from my compound to take me Fajara for free treatment and brought me back. I was not even married at the time. Fajara MRC is a good place.	Interview	Farmer	Malaria Transmission Dynamics
We know the MRC only as a healthcare provider.	Community Sensitization	Farmer	Malaria Transmission Dynamics
As far as I'm concerned, when it comes to the MRC issue, I respect them and am happy with them. I only feel happiness because I have seen the benefit. My granddaughter was seriously sick. No one thought she will recover but when we entered MRC, they would come to collect her and bring her back till she regained her health.	Interview	Old woman	Prinogam
Some time back, my brother fell sick in my hands. For two years his body was completely weak and eventually died. (…) It was the MRC who came and picked up my brother and took him to Bansang and from there he was taken to Banjul where he died. Whether he survived or not, I was relieved from the burden of carrying him. It was MRC who helped me to do that.	Interview	Farmer	Prinogam
About the MRC, we all say that they are good at curing people, because even me sitting down here, if I have the opportunity to join the MRC I would be very grateful. The reason is that there is good health in it. If someone gets sick and goes there, they treat you well, give you proper medicines till you get well.	Interview	Housewife/ farmer	Prinogam
**Quotes on barriers to research participation**			
*Interviewer: Why would you not like to participate in the study the MRC is about to start*. R: I do not know. I do not intend to participate, that is my opinion. I am hungry, I would like to have food. I am too old.	Interview	Housewife/farmer	Malaria Transmission Dynamics
I was participating in the program grant (i.e. Malaria Transmission Dynamics study), until my husband saw my participation identification card and told the whole compound to stop participating without giving a reason.	Interview	Housewife	Malaria Transmission Dynamics
It is my husband who brought me here. So if he said I should withdraw from the program (i.e. Malaria Transmission Dynamics study), I must withdraw.	Interview	Housewife	Malaria Transmission Dynamics study
My husband is in Europe, I will tell him and wait for his consent to join or not.	Interview	Housewife	Prinogam
*Interviewer: Why don't you want to participate in the future study?* R: I will be travelling soon. I don't know when I will come back.	Interview	Housewife	Prinogam
*I: Do people think that they are trying to sell blood?* R: For us we don't know that. When we give them our blood, we do so for them to check but after that what happens to the blood, we cannot tell. We don't know that.*I: And what do you think happens to the blood after the collection? Don't they tell you the reason why they are taking your blood?* R: What they say is that they are going to check it. They say that they are going to look for the disease.*I: So personally that is what you believe?* R: (The old woman adds) Only God knows, we have no idea. Whether what they are saying is true or false, only God knows it.	Group discussion	Housewife/ farmer	Prinogam
R: Some people think that the MRC only collect the blood and use it for their own businesses; I personally don't believe this. *I: Do you mean selling out the blood or that people could use it for something bad, like witchcraft?* R: Yes, that is what some people think. In fact, when you get to certain villages some people did not join their (MRCG research) programs. Even here in the village some people refused because they think their blood will be sold.	Interview	Farmer	Malaria Transmission Dynamics

#### 3.6.2 Barriers to research participation

The perceived barriers to research participation among the non‐participants were travelling, old age, sickness, reluctance to give a blood sample and the disapproval of the household head (Table 14). For people who were undecided about their participation, critical factors that would influence their decision were the amount of blood to be taken, the agreement of the household head and the potential benefits of participation. Qualitative research during both studies confirmed that the approval of the compound head or husband was an important factor for the decision‐making of other people in the compound or household (Table 14). In rural Gambia, the male compound or household head is an influential figure. Qualitative research carried out after the community meetings of both studies showed that some people were concerned about blood samples collected in clinical research because of the fear that blood might be sold or taken by MRCG, or that too much blood loss would lead to loss of strength, which is associated with depleting life‐force (Table 14).

### 3.7 Perceptions of MRCG

Qualitative research before and after the community meetings of both studies showed that the perceived benefits and disadvantages of participating were related to (i) the reputation of the MRCG in the Gambia and (ii) previous personal experiences of participating in medical research as well as (iii) experiences of relatives and other acquaintances. Of all respondents, 37.1% had previously participated in MRCG research projects (Table 5). When asked if they could remember the purpose of the project they had been part of, some did not remember while others mentioned the objective of the research (malaria vaccination, bed nets, pneumococcal vaccination, tuberculosis, polio) or described the necessity of those projects to collect blood: *‘they used to come here and take me along with my twin brother to test our blood’* or ‘*it was only about blood checking’*. Still others described the benefits they received, for example *‘my child was enrolled with the MRC, they used to give us free medication’* or *‘since I was young they were taking care of me: giving me treatment and medication’*. People who decided not to participate in previous MRCG studies claimed they were travelling too much or that the MRCG was *‘not popular in the past’*. Qualitative data showed that these considerations about MRCG and recollections about previous research done by MRCG influenced people's decision‐making, contributing to the discrepancy between people's attendance of the community meeting (38.7%) (Table 7), and the overall willingness to participate prior to individual consent (91.1%) (Table 9).

## 4 DISCUSSION

Community sensitization is often proposed as a necessary strategy to ensure the success of medical interventions and research programs, by both research staff and community members and it is often part of the procedures aiming to improve the community engagement and the informed consent process. Little is known however about the impact community sensitization has on individual decision‐making for trial participation. Our study presents three interrelated key findings and potential challenges to this process.

First, although only 38.7% of all respondents were present at the *Prinogam* community sensitization meetings, 92.7% of all respondents were aware of the trial when surveyed *after* the community meeting and *before* individual screening for study participation and individual informed consent. It looks like the first‐hand, accurate information delivered at the community meetings was cascaded as ‘lay’ information to those other community members who could not attend the sessions.

Second, this information seems to influence the decision to participate in the trial as 91.1% of respondents stated their intention to participate in the study. In stark contrast, respondents who were completely unaware of the trial were less likely to be willing to participate. These findings should be further confirmed, since the study was conducted before the start of the trial's recruitment thus we do not know the actual outcome of consent interviews. However, these observations in The Gambia are in line with previous research in Burkina Faso where parents decided to enroll their children in research *before* the individual informed consent process, and with similar anecdotal observations in other sub‐Saharan African research settings.^15^ However, neither the study in Burkina Faso nor the subsequent anecdotal observations had looked at the potential impact of community sensitization on individual consent. In our study, it seems that the community sensitization has positively oriented the individual decision making, but it remains unclear to what extent it has improved the understanding of the nature and the objectives of the research.

Third, research participation was additionally driven by reasons already documented in other sub‐Saharan African contexts, such as trust in the research institution and the awareness that they would bring good healthcare, but also receiving free good‐quality drugs and additional benefits, such as free transportation to the health care facilities.^16^ These expectations are often based on the individual and community's previous research experience and the reputation of the local research institution.

This study also highlighted the complexity of reaching ‘the community’ through sensitization activities, since it is not possible to get all households represented at the meetings. In addition, engagement with community members always implies engaging with existing social relations and hierarchies.^17^ Some village members might have been absent from the meetings due to frictions with other community members. For certain compounds, the approval of the male head of the compound or household may be key for women and children's research participation. Prevailing international perspectives on informed consent emphasize the importance of individual decision‐making, but in many settings community leaders or family members *de facto* play at least a minor role in the decision‐making process for participation in research.^18^

### 4.1 Study limitations

A limitation of the quantitative study was the non‐random sample. Although the target population for the survey was compound heads of all compounds in the study villages, about 50% of the respondents were other household members. In addition, the willingness to participate as stated prior to the studies could not be contrasted with actual participation data. Further research could include a follow up to demonstrate whether reported willingness or unwillingness to participate correlates with actual research participation, and a further check of whether attendance at community sensitization meeting or receiving “cascaded” information consistently improves the understanding and long‐term recall of each specific research.

## 5 CONCLUSION

Although not substituting individual consent, community sensitization meetings represent a first step in getting access to the communities. In practice, not everyone may be present during these meetings, but key figures within the compound are more likely to be present and explain the study to compound members, acquaintances and friends afterwards. Therefore, “community meetings” may be seen as an effective tool in providing first‐hand, reliable information to communities as the information is cascaded to those who could not attend the sessions. Further research is needed to assess how the informal spread of information further shapes people's expectations, how the process engages with existing social relations and hierarchies (e.g. local political power structures; permissions of heads of households) and how this influences or changes individual consent. In addition, more research is needed to understand if mechanisms similar to those observed in The Gambia may be expected in research‐naïf communities.

## CONFLICT OF INTEREST

No conflicts declared.

## Notes

1 WMA. *Declaration of Helsinki ‐ Ethical Principles for Medical Research Involving Human Subjects*. 2013; Research TNC for the P of HS of B and B. *The Belmont Report: Ethical Principles and Guidelines Ofr the Protection of Human Subjects of Research*. 1979; Nuffield Council on Bioethics. *The Ethics of Research Related to Healthcare in Developing Countries*. 2000; CIOMS. *International Ethical Guidelines for Health‐Related Research Involving Humans*. *Volume 2008*. Geneva: WHO Press; 2016(February).

2 Afolabi MO et al. Informed consent comprehension in African research settings. *Trop Med Int Heal* 2014, 19:625–642; Chima SC. Evaluating the quality of informed consent and contemporary clinical practices by medical doctors in South Africa: An empirical study. *BMC Med Ethics* 2013, 14(Suppl 1):S3.

3 Paré Toe L et al. Could the decision of trial participation precede the informed consent process? Evidence from Burkina Faso. *PLoS One* 2013, 8:e80800; Peeters Grietens K, et al. Doctors and Vampires in Sub‐Saharan Africa: Ethical Challenges in Clinical Trial Research. *Am J Trop Med Hyg* 2014, 91:213–215.

4 Afolabi MO et al., op cit. note 2, p. 3; Marshall PM: *Ethical Challenges in Study Design and Informed Consent for Health Research in Resource‐Poor Settings*. *Volume 5*; 2007.

5 Gikonyo C et al. Taking social relationships seriously: Lessons learned from the informed consent practices of a vaccine trial on the Kenyan Coast. *Soc Sci Med* 2008, 67:708–720; Lang TA et al. Approaching the community about screening children for a multicentre malaria vaccine trial. *Int Health* 2012, 4:47–54.

6
*Work for Human Development*. 2015.

7 O'Neill S et al. Foul wind, spirits and witchcraft: illness conceptions and health‐seeking behaviour for malaria in the Gambia. *Malar J* 2015, 14:167.

8 Ibid; O'Neill S et al. The Importance of Blood Is Infinite: Conceptions of Blood as Life Force, Rumours and Fear of Trial Participation in a Fulani Village in Rural Gambia. *PLoS One* 2016:1–13; Dierickx S et al.: Factors Associated with Non‐Participation and Non‐Adherence in Directly Observed Mass Drug Administration for Malaria in The Gambia. *PLoS One* 2016, 11:e0148627.

9 Dierickx S et al., *op cit*. note 8 p. 7; Mwesigwa J et al. On‐going malaria transmission in The Gambia despite high coverage of control interventions: a nationwide cross‐sectional survey. *Malar J* 2015, 14:314.

10 Okebe J et al. The gametocytocidal efficacy of primaquine in malaria asymptomatic carriers treated with dihydroartemisinin‐piperaquine in The Gambia (PRINOGAM): study protocol for a randomised controlled trial. *Trials* 2015, 16:70; Afolabi MO et al. A multimedia consent tool for research participants in the Gambia: A randomized controlled trial. *Bull World Health Organ* 2015, 93:320–328A.

11 Afolabi MO et al. *op. cit*. note 10, p. 8

12 Tashakkori A, Teddlie C. 1997. *Mixed‐Methodology: Combining Qualitative and Quantitative Approaches*. London: Sage Publications ltd.

13 Ibid.; Coyne IT. Sampling in qualitative research. Purposeful and theoretical sampling; merging or clear boundaries? *J Adcance Nurs* 1997, 26:623–630.

14 Jupp V. 2006. *The SAGE Dictionary of Social Research Methods*. London: Sage Publications ltd.

15 Paré Toe L et al., *op cit*. note 3, p. 4; Ravinetto RM et al. Participation in medical research as a resource‐seeking strategy in socio‐economically vulnerable communities: Call for research and action. *Trop Med Int Heal* 2015, 20:63–66.

16 Fairhead J, Leach M, Small M. Where techno‐science meets poverty: Medical research and the economy of blood in The Gambia, West Africa. *Soc Sci Med* 2006, 63:1109–1120; Kingori P. Experiencing everyday ethics in context : Frontline data collectors perspectives and practices of bioethics. *Soc Sci Med* 2013, 98:361–370; Ravinetto R et al. Health research: the challenges related to ethical review and informed consent in developing countries. *Goirnale Ital di Med Trop* 2010, 15:15–20; Molyneux CS, Peshu N, Marsh K. Trust and informed consent: insights from community members on the Kenyan coast. *Soc Sci Med* 2005, 61:1463–1473; Lock M, Nguyen V‐K.2010. *An Anthropology of Biomedicine*.

17 Kamuya D, Marsh V, Molyneux S. What we learned about voluntariness and consent: incorporating “background situations” and understanding into analyses. *Am J Bioeth* 2011, 11:31–3; Marsh VM et al. Working with concepts: The role of community in international collaborative biomedical research. *Public Health Ethics* 2011, 4:26–39; Tarimo EAM et al. Reasons for Declining to Enroll in a Phase I and II HIV Vaccine Trial after Randomization among Eligible Volunteers in Dar es Salaam, Tanzania. *PLoS One* 2011, 6:e14619; Krogstad DJ et al. Informed consent in international research: The rationale for different approaches. *Am J Trop Med Hyg* 2010, 83:743–747; Tindana PO et al. Aligning community engagement with traditional authority structures in global health research: A case study from Northern Ghana. *Am J Public Health* 2011, 101:1857–1867.

18 Marshall PM, op.cit. note 4, p. 4.

